# Teenage pregnancy: the impact of maternal adolescent childbearing and older sister’s teenage pregnancy on a younger sister

**DOI:** 10.1186/s12884-016-0911-2

**Published:** 2016-05-25

**Authors:** Elizabeth Wall-Wieler, Leslie L. Roos, Nathan C. Nickel

**Affiliations:** Manitoba Centre for Health Policy, Department of Community Health Sciences, Faculty of Health Sciences, College of Medicine, University of Manitoba, 408-727 McDermot Avenue, Winnipeg, Manitoba R3E 3P5 Canada

**Keywords:** Teenage pregnancy, Familial influence, Social modelling, Intergenerational effects, Linkable administrative data

## Abstract

**Background:**

Risk factors for teenage pregnancy are linked to many factors, including a family history of teenage pregnancy. This research examines whether a mother’s teenage childbearing or an older sister’s teenage pregnancy more strongly predicts teenage pregnancy.

**Methods:**

This study used linkable administrative databases housed at the Manitoba Centre for Health Policy (MCHP). The original cohort consisted of 17,115 women born in Manitoba between April 1, 1979 and March 31, 1994, who stayed in the province until at least their 20^th^ birthday, had at least one older sister, and had no missing values on key variables. Propensity score matching (1:2) was used to create balanced cohorts for two conditional logistic regression models; one examining the impact of an older sister’s teenage pregnancy and the other analyzing the effect of the mother’s teenage childbearing.

**Results:**

The adjusted odds of becoming pregnant between ages 14 and 19 for teens with at least one older sister having a teenage pregnancy were 3.38 (99 % CI 2.77–4.13) times higher than for women whose older sister(s) did not have a teenage pregnancy. Teenage daughters of mothers who had their first child before age 20 had 1.57 (99 % CI 1.30–1.89) times higher odds of pregnancy than those whose mothers had their first child after age 19. Educational achievement was adjusted for in a sub-population examining the odds of pregnancy between ages 16 and 19. After this adjustment, the odds of teenage pregnancy for teens with at least one older sister who had a teenage pregnancy were reduced to 2.48 (99 % CI 2.01–3.06) and the odds of pregnancy for teen daughters of teenage mothers were reduced to 1.39 (99 % CI 1.15–1.68).

**Conclusion:**

Although both were significant, the relationship between an older sister’s teenage pregnancy and a younger sister’s teenage pregnancy is much stronger than that between a mother’s teenage childbearing and a younger daughter’s teenage pregnancy. This study contributes to understanding of the broader topic “who is influential about what” within the family.

## Background

The risks and realities associated with teenage motherhood are well documented, with consequences starting at childbirth and following both mother and child over the life span.

Teenage births result in health consequences; children are more likely to be born pre-term, have lower birth weight, and higher neonatal mortality, while mothers experience greater rates of post-partum depression and are less likely to initiate breastfeeding [1, 2]. Teenage mothers are less likely to complete high school, are more likely to live in poverty, and have children who frequently experience health and developmental problems [3]. Understanding the risk factors for teenage pregnancy is a prerequisite for reducing rates of teenage motherhood. Various social and biological factors influence the odds of teenage pregnancy; these include exposure to adversity during childhood and adolescence, a family history of teenage pregnancy, conduct and attention problems, family instability, and low educational achievement [4, 5].

Mothers and older sisters are the main sources of family influence on teenage pregnancy; this is due to both social risk and social influence. Family members both contribute to an individual’s attitudes and values around teenage pregnancy, and share social risks (such as poverty, ethnicity, and lack of opportunities) that influence the likelihood of teenage pregnancy [6, 7]. Having an older sister who was a teen mom significantly increases the risk of teenage childbearing in the younger sister and daughters of teenage mothers were significantly more likely to become teenage mothers themselves [8, 9]. Girls having both a mother and older sister who had teenage births experienced the highest odds of teenage pregnancy, with one study reporting an odds ratio of 5.1 (compared with those who had no history of family teenage pregnancy) [5]. Studies consistently indicate that girls with a familial history of teenage childbearing are at much higher risk of teenage pregnancy and childbearing themselves, but methodological complexities have resulted in inconsistent findings around “parent/child sexual communication and adolescent pregnancy risk” [10]. A review of family relationships and adolescent pregnancy risk found risk factors to include living in poor neighborhoods and families, having older siblings who were sexually active, and being a victim of sexual abuse [10]. Research around the impact of sister’s teenage pregnancy has been limited to mostly qualitative studies using small samples of minority adolescents in the United States [5, 11].

To our knowledge, no previous studies have examined the impact of an older sister’s teenage pregnancy on the odds of her younger sister having a teenage pregnancy, and compared this effect with the direct effect of having a mother who bore her first child before age 20. By controlling for a variety of social and biological factors (such as neighborhood socioeconomic status, marital status of mother, residential mobility, family structure changes, and mental health), and the use of a strong statistical design—propensity score matching with a large population-based dataset—this study aims to determine whether teenage pregnancy is more strongly predicted by having an older sister who had a teenage pregnancy or by having a mother who bore her first child before age 20.

## Methods

### Setting

The setting of this study, Manitoba, is generally representative of Canada as a whole, ranking in the middle for several health and education indicators [12, 13]. At the time of the 2011 Census, approximately 1.2 million people resided in Manitoba, with more than half (783,247) living in the two urban areas, Winnipeg and Brandon [14]. Teenage pregnancy rates in Manitoba exceed the national; in 2010 teenage pregnancy rates in Canada were 28.2 per 1000, in Manitoba the rate was 48.7 per 1000 [15]. The Manitoba teen pregnancy rates in 2010 were slightly lower than rates in England and Wales (54.6 per 1000), and the United States (57.4 per 1000) [16, 17].

### Data

The Manitoba Population Health Research Data Repository contains province-wide, routinely collected individual data over time (going back to 1970 in some files), across space (with residential location documented using six digit postal codes), for each family (with changes in family structure recorded every 6 months) and for each resident. Health variables are measured continuously from physician claims and hospital abstracts (as long as an individual remains in Manitoba) [18].

A research registry identifies every provincial resident, with information on births, arrival and departure dates, and deaths created from the provincial health registry and coordinated with Vital Statistics files. Given approximately 16,000 births annually, follow-up (about 74 % over 20 years) is comparable to that in the largest cohort studies based on primary data [19]. Previous research using similar data shows the results are not biased by individuals leaving the province or dying. Information on data linkage, confidentiality/privacy, and validity of the datasets used have been described elsewhere [20–22]. Children are linked to mothers using hospital birth record information; the mother was noted in essentially all cases [23]. Sisters were defined as having the same biological mother.

**Fig. 1:**
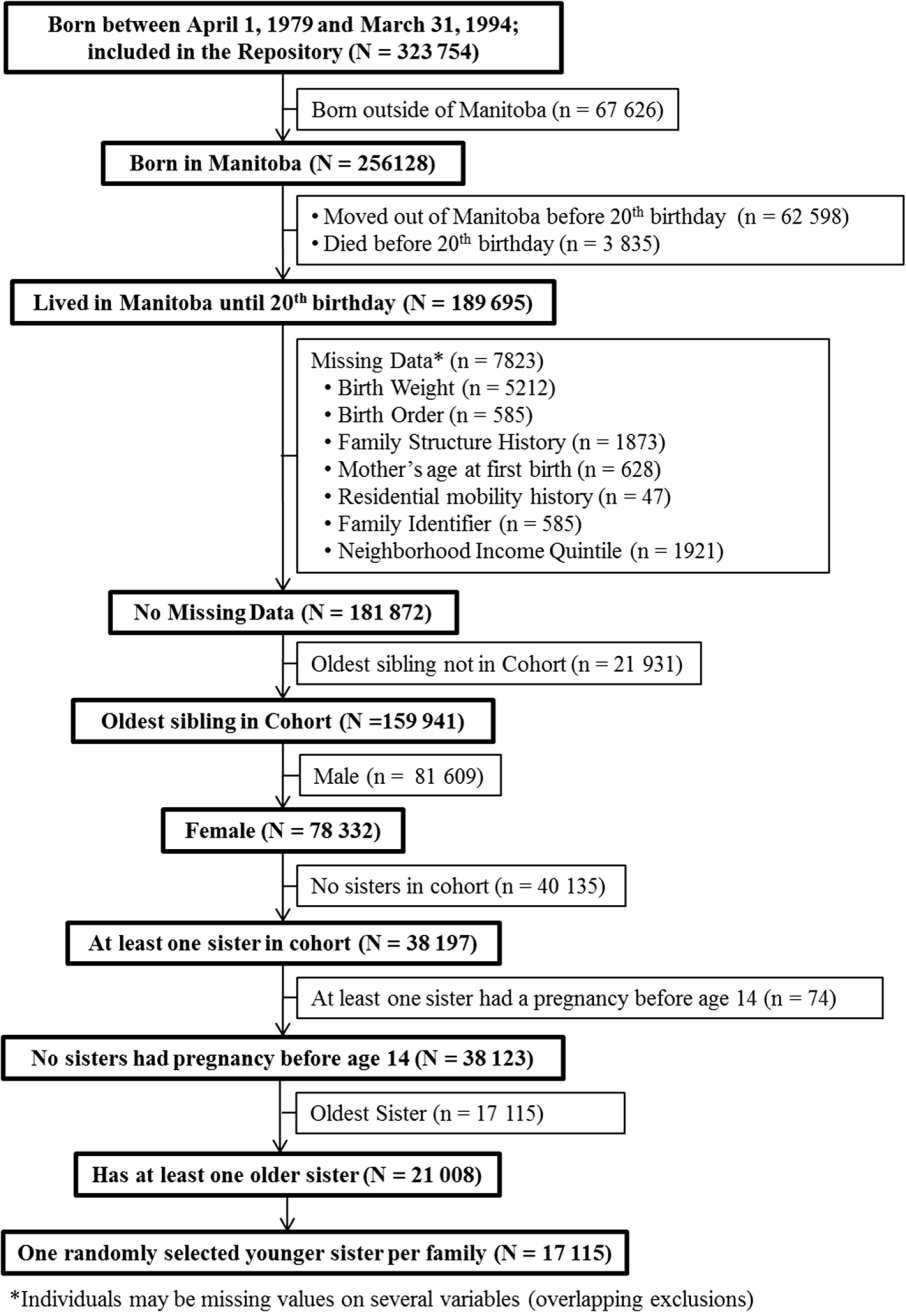
Cohort selection

The cohort consists of women who were born in Manitoba between April 1, 1979 and March 31, 1994, stayed in the province until at least their 20^th^ birthday, had at least one older sister, and had no missing values on key variables. In this study, teenage pregnancies are defined as those between the ages of 14 and 19; pregnancies prior to age 14 were excluded due to low numbers and for comparability to other studies. For this reason, families in which at least one sister had a pregnancy before age 14 were removed (34 families). To address threats of independence, when a family had more than one younger sister (more than two daughters), one younger sister was randomly selected. Figure 1 diagrams the selection trajectory for the 17,115 individuals selected—boxes in bold indicate the included cohort. At age 14, just over 85 % of girls in this cohort were living in the same postal code as at least one older sister.

#### Outcome

Teenage pregnancy was defined as having at least one pregnancy between the ages of 14 and 19 (inclusive). A pregnancy is defined as having at least one hospitalization of with a live birth, missed abortion, ectopic pregnancy, abortion, or intrauterine death, or at least one hospital procedure of surgical termination of pregnancy, surgical removal of ectopic pregnancy, pharmacological termination or pregnancy or intervention during labour and delivery. Pregnancy status was determined by ICD-9-CM codes (for diagnoses before April 1, 2004), ICD-10-CA codes (for diagnoses on or after April 1, 2004), and Canadian Classification of Health Intervention (CCI) codes in the hospital discharge abstract database [24]. Appendix 1 presents specific codes used to determine pregnancy status.

#### Independent variable

The independent variables of interest were whether an individual had an older sister with a teenage pregnancy (defined for all sisters as described above) and whether an individual’s mother bore her first child before age 20.

#### Covariates

Based on an extensive literature review and availability of information in the database, several key variables describing neighborhood, maternal, and individual characteristics were included [4, 25]. Covariates measure characteristics in the younger sister’s life before age 14. Neighborhood socioeconomic status at age 14 was measured by the Socioeconomic Factor Index (SEFI) (higher SEFI score corresponds with lower socioeconomic status), which is generated using Manitoba (Statistics Canada) dissemination areas [26]. This index combines neighborhood information on income, education, employment, and family structure. These neighborhoods typically include between 400 and 700 urban individuals and are somewhat larger in rural areas. Neighborhood location at age 14 was divided into urban (Winnipeg and Brandon), rural south (South Eastman, Central, and Assiniboine Regional Health Authorities), and rural mid/north (North Eastman, Interlake, Parkland, Nor-Man, Churchill, and Burntwood Regional Health Authorities). The maternal characteristic included is marital status at birth of child. An individual’s number of older sisters was also accounted for.

Three time-varying covariates between birth and age 13 for the younger sister were included in the study- mental health conditions, residential mobility, and family structure change. These variables can occur at specific points in time and the timing of their occurrence can differ across individuals. Mental health is defined using the Johns Hopkins University Adjusted Clinical Group (ACG) software; this software groups medical and hospital diagnoses over the course of a year into 27 Major Expanded Diagnostic Clusters (MEDCs) [27]. If for 1 year between birth and age 13, the diagnoses an individual received fell into the ‘Mental Health’ MEDC, that individual was categorized as having mental health conditions before age 13. Residential mobility was measured by at least one residential move (defined by change in six digit postal code) between birth and age 13. At least one change in family structure (parental divorce, death, marriage, remarriage) between birth and age 13 was noted as ‘family structure change’.

Low educational achievement has been linked to an increased risk of teenage pregnancy [28]. The earliest measure of educational achievement available is the Grade 9 Achievement Index, which was built on a technique developed by Mosteller and Tukey using enrollment files, course grades, and the provincial population registry [29, 30]. As some of the individuals in this cohort experience their first pregnancy before completing grade 9, this covariate is only appropriate for girls having their first pregnancy after their 16^th^ birthday. Sensitivity testing was done with this population to determine how strongly educational achievement affected the odds of the variables of interest.

### Analytic approach

**Fig. 2:**
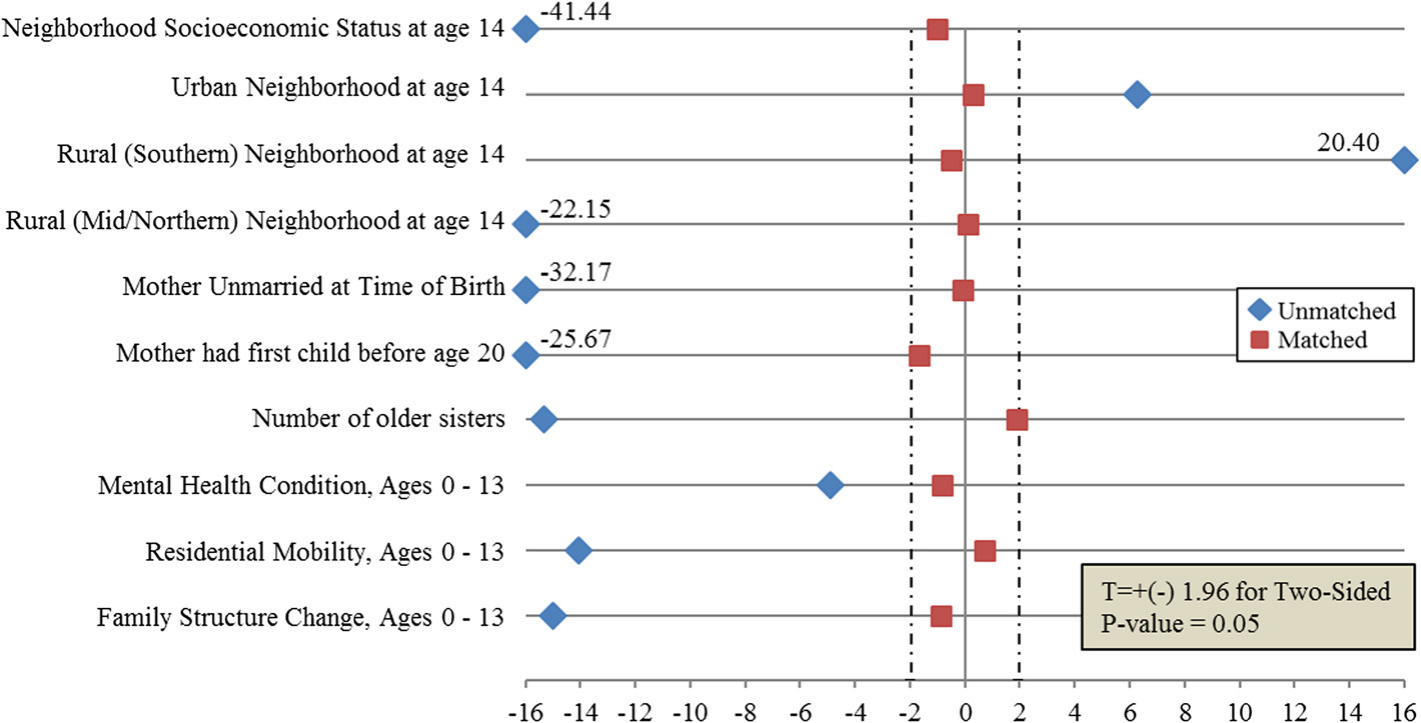
Checking covariate balance of older sister’s teenage pregnancy status

**Fig. 3:**
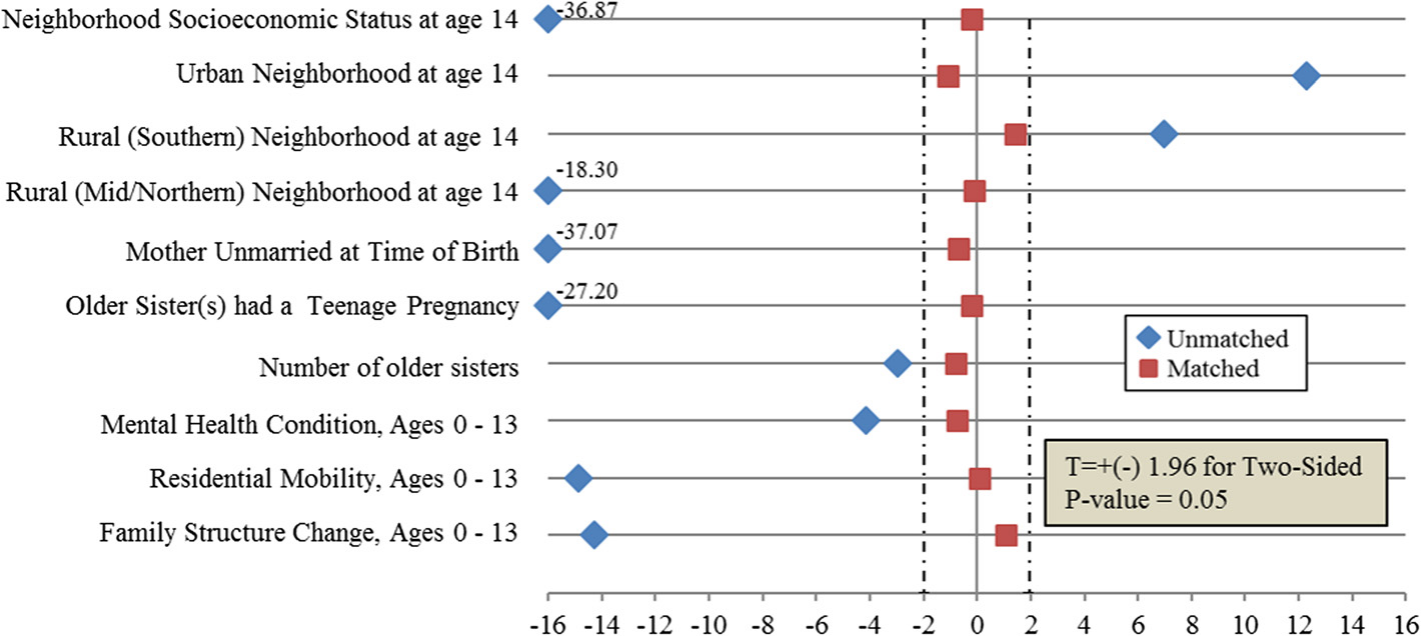
Checking covariate balance of mother’ teenage mom status

The relationship between pregnancy during one’s teenage years and having an older sister who became pregnant during adolescence or having a mother who bore her first child as a teenager is confounded by many measured and unmeasured characteristics. We adjusted for these confounding characteristics using 2:1 propensity score matching [31]; two controls were matched with every case as this “will result in optimal estimation of treatment effect [32]”. Propensity score matching both enables adjustment for several confounders simultaneously and facilitates diagnostic tests to identify whether the adjustment strategy created comparable exposure groups (i.e., whether women with and without an older sister who got pregnant during adolescence are similar on observed characteristics) [31]. Logistic regression models were used to calculate propensity scores for two responses—the predicted probability of having an older sister having a teenage pregnancy and the predicted probability of having a mother bearing her first child before age 20. For each model, we investigated the comparability of our two groups—those with and without an older sister having a teenage pregnancy, and those with and without a mother who bore her first child as a teenager—using two diagnostics. A kernel density plot verified that the distribution of propensity scores in our two groups overlapped [33]; each case was matched to two controls using greedy matching [34]. Second, after matching, the balance of the covariates was assessed using standard differences and t-tests. Covariate balance was checked by t-statistics calculated for the standardized differences between cases and controls for each covariate before and after matching. Any point outside of the two vertical dotted lines signified a statistically significant difference between the cases and controls on that covariate (at *p* = 0.05) (Figs. 2 and 3).

Conditional logistic regression analysis of the matched cohorts examined the impact of an older sister’s teenage pregnancy and of a mother’s teenage childbearing on teenage pregnancy. Sensitivity analysis helped assess the validity of the assumption of no unobservable confounders, and assessed how strong the influence of unobserved covariates would have to be in order to nullify our findings [35, 36]. The lower limit of the 99 % confidence interval (selected to be more conservative) was used to determine the threshold unobserved covariates would have to reach to void the observed relationship.

## Results

### Impact of older sister having a teenage pregnancy

Table 1 displays the descriptive statistics of the covariates and outcome variables. Of the girls having an older sister with a teenage pregnancy, 40.4 % had a teenage pregnancy. This is significantly higher than the 10.3 % teenage pregnancy rate among those not having an older sister with a teenage pregnancy.

**Table 1:** Covariates and outcomes (older sister having a teenage pregnancy)

	Older sister did not have a teenage pregnancy (*n* = 13,624)	Older sister had a teenage pregnancy (*n* = 3491)
	Mean/proportion	Mean/proportion
Outcome		
Teenage pregnancy	0.103	0.404
Time invariant covariates		
Neighborhood socioeconomic status (SEFI) at age 14^a^	−0.109	0.754
*Location of neighborhood at age 14*		
Urban	0.51	0.45
Rural South	0.275	0.134
Rural Mid/North	0.215	0.416
Mother unmarried at time of birth	0.144	0.43
Mother had first child before age 20	0.085	0.292
Number of older sisters	1.086	1.228
Time-varying covariates, ages 0–13		
Mental health condition	0.156	0.192
Residential mobility	0.558	0.684
Family structure change	0.156	0.279

^a^NOTE: Higher Socioeconomic Factor Index (SEFI) corresponds with lower SES

The covariates, in general, accord with social stratification theory [37]. Teens with an older sister having a teenage pregnancy were also more likely to have been born to an unmarried mother and have a mother who herself was a teenage mother (43 % versus 14 %). At age 14, approximately 42 % of those whose older sister had a teenage pregnancy lived in Rural Mid/Northern Manitoba; only 22 % of those whose older sister did not have a teenage pregnancy lived in this region at age 14. Lower teenage pregnancy was associated with residence in relatively prosperous southern Manitoba. Individuals with older sisters having teenage pregnancies were more likely to live in lower socioeconomic status neighborhood (higher SEFI scores at age 14) with higher rates of residential mobility (68 % vs 59 %), family structure change (28 % vs 16 %), and mental health issues (19 % vs 16 %).

After propensity score matching (on all variables in Fig. 2), the final sample consisted of 1873 cases and 3746 controls (1:2); a total of 1618 cases and 9878 controls were excluded from the analysis. T-statistics calculated for each covariate before and after matching to check for covariate balance; all covariates differed significantly in the unmatched sample and balanced in the matched sample (Fig. 2).

The final conditional logistic regression model indicates the odds of becoming pregnant before age 20 for those having an older sister with a teenage pregnancy to be 3.38 (99 % CI 2.77–4.13) times greater than for girls whose older sister(s) did not have a teenage pregnancy (Table 3).

### Impact of mother’s teenage childbearing

Table 2 displays the descriptive statistics of the covariates and outcome variables. Of the girls having a teenage mother, 39.4 % had a teenage pregnancy. This is significantly higher than the 13.1 % teenage pregnancy rates among those whose mother bore her first child after age 19.

**Table 2:** Covariates and outcomes (mother’s teenage childbearing)

	Mother had first child after age 19 (*n* = 14934)	Mother had first child before age 20 (*n* = 2181)
	Mean/proportion	Mean/proportion
Outcome		
Teenage pregnancy	0.131	0.394
Time invariant covariates		
Neighborhood socioeconomic status (SEFI) at age 14^a^	−0.052	1.138
*Location of neighborhood at age 14*		
Urban	0.515	0.375
Rural South	0.255	0.191
Rural Mid/North	0.230	0.434
Mother unmarried at time of birth	0.150	0.559
Older sister(s) had a teenage pregnancy	0.165	0.468
Number of older sisters	1.112	1.138
Time-varying covariates, ages 0–13		
Mental health condition	0.158	0.196
Residential mobility	0.564	0.719
Family structure change	0.162	0.31

^a^NOTE: Higher Socioeconomic Factor Index (SEFI) corresponds with lower SES

After propensity score matching (on all variables in Fig. 3), the final sample consisted of 1522 cases and 3044 controls (1:2); a total of 659 cases and 11890 controls were excluded from the analysis. T-statistics calculated for each covariate showed all covariates to differ significantly in the unmatched sample and to balance in the matched sample (Fig. 3).

The final conditional logistic regression model indicates that the odds of becoming pregnant before age 20 for those whose mother had her first child before age 20 are 1.57 (99 % CI 1.30–1.89) times greater than for girls whose mother had her first child after age 19 (Table 3). Thus, the impact of being born to a mother having her first child before age 20 on teenage pregnancy is much less than that of an older sisters’ teenage pregnancy.

**Table 3:** Odds ratios for original and additional analyses

	Without adjusting for educational achievement			Adjusting for educational achievement		
	OR	99 % CI	N	OR	99 % CI	N
Older sister had a teenage pregnancy	3.38	2.77–4.13	5619	2.48	2.01–3.06	5163
Mother had first child before age 20	1.57	1.30–1.89	4566	1.39	1.15–1.68	4487

### Sensitivity analysis and limitations

With the confidence interval for the first model (examining the association between an older sister’s teenage pregnancy and a younger sister’s teenage pregnancy) ranging between 2.77 and 4.13, to attribute the higher rates of teenage pregnancy to unmeasured confounding rather than to an older sisters’ teen pregnancy status, that covariate would need to generate more than a 2.8-fold increase in the odds of teenage pregnancy and be a near perfect predictor of teenage pregnancy. In the second model (assessing the association between a mother’s teenage childbearing and a younger sister’s teenage pregnancy), the 99 % confidence interval was 1.30 to 1.89; unobserved covariates would need to produce a much smaller increase in odds of teen pregnancy to nullify this finding.

Although linkable administrative data have significant advantages, some important predictors are lacking. Information on involvement with Child and Family Services (CFS) and parental use of income assistance have recently been added to the Manitoba databases, but do not cover the cohort used here. While having a teenage mother and becoming a teenage mother have both been linked to involvement with CFS, in 2001 less than two percent of children under age 18 were in care [38, 39]. A variable available (and applicable) for a subpopulation is educational achievement, which is highly correlated with both involvement with CFS and parental welfare use [40]. These two new measures would likely explain little additional variance in teenage pregnancy. Appendix 2 describes the cohort and propensity score matching for this additional analysis, comparing these findings with the original results in Table 3. Educational attainment is measured using the Grade 9 Achievement Index, a standardized measure taking into account the number of courses completed in Grade 9 and the average marks of those courses. After adjusting for educational achievement, the odds of teenage pregnancy for teens with at least one older sister who had a teenage pregnancy were reduced to 2.48 (99 % CI 2.01–3.06) and the corresponding odds for teen daughters of teenage mothers were lowered to 1.39 (99 % CI 1.15–1.68).

## Discussion

The rate differences of teenage pregnancy were similar for those whose older sister had a teenage pregnancy (40.4 per 100 - 10.3 per 100 = 30.1 per 100) and for those whose mother bore her first child before age 20 (39.4 per 100 - 13.1 per 100 = 26.3 per 100). After propensity score matching on a series of variables, the odds of becoming pregnant for a teenager were much higher if her older sister had a teenage pregnancy than if her mother had been a teenage mother. For both older sisters’ teenage pregnancy and mother’s teenage childbearing, the odds in this study are lower than those reported elsewhere; this is likely due to the larger sample size, more rigorous methods, and inclusion of important predictors.

Several examinations of family histories in the literature show older sisters to have the greatest influence on a younger sister’s odds of having a teenage pregnancy. Controlling for family socioeconomic status, maternal parenting, and sibling relationships, teens with an older sister who had a teenage birth were 4.8 times more likely to have a teenage birth themselves; these odds increased to 5.1 if both the older sister and mother had a teenage birth [11]. Four older studies estimated the rate of teen pregnancy to be between 2 and 6 times higher for those with older sisters having a teenage pregnancy [41]. This work focused primarily on young black women in the United States and controlled for limited confounders (aside from race and age). None of the previous studies examining the impact of an older sister’s teenage pregnancy controlled for mother’s teenage childbearing or time-varying factors before age 14 (mental health, residential mobility, family structure changes); this research probably overestimated the relationship between sisters’ teenage pregnancy status.

The mechanisms driving the relationship between an older sister’s teenage pregnancy and the pregnancy of a younger adolescent sister have been examined through approaches based on social learning theory, shared parenting influences, and shared societal risk [41]. Bandura’s social learning theory indicates that “most human behavior is learned observationally through modeling: from observing others one forms an idea of how new behaviors are performed, and on later occasions this coded information serves as a guide for action” [7]. When sisters live in the same environment, seeing an older sister go through a teenage pregnancy and childbirth may make this a more acceptable option for the younger sister [11]. Not only do both sisters have the same maternal influence that may affect their odds of teenage pregnancy, having an older sister who is a teenage mother may change the parenting style of the mother. Mothers involved in parenting of their teenage daughters’ child may have “supervised their children less, communicated with their children less about sex and contraception, and perceived teenage sex as more acceptable when the older daughter’s status changed from pregnant to parenting” [42]. Finally, both sisters share the same social risks, such as poverty, ethnicity, and lack of opportunities, that increase their chances of having a teenage pregnancy [42].

Having a mother bearing her first child before age 20 was a significant predictor for teenage pregnancy. We found daughters of teenage mothers to be 51 % more likely to have a teenage pregnancy than those whose mothers were older than 19 when they bore their first child. This is quite close to the 66 % increase found by Meade et al (2008), who controlled for many of the same variables except having an older sister with a teenage pregnancy, and the time-varying covariates of family structure change, mental health conditions, and residential mobility. Meade et al. [9] did adjust for school performance; in the adjusted sub-sample, the odds ratio reduced to 1.34, indicating a 34 % increase in teenage pregnancy.

Intergenerational teenage pregnancy may be influenced by such mechanisms as “biological heritability, intergenerational transmission of values regarding family, the mother’s level of fertility, the indirect impact of socioeconomic and family environment through educational deficits or low opportunity or aspirations, and directly through the mother’s role modeling” [43]. Women bearing their first child in their adolescence are more likely to pass on “risky” characteristics, which could produce negative outcomes in their offspring [44]. Another mechanism identified as contributing to intergenerational teenage pregnancy is that daughters of teenage mothers have an increased internalized preference for early motherhood, have low levels of maternal monitoring, and are thus more likely to become sexually active at a young age and engage in unprotected sex [44]. The influence of a mother’s teenage pregnancy therefore works through the environment created and parenting style assumed as a result of a mother’s teenage childbearing.

The use of administrative data to conduct health services research has some significant advantages and limitations. Administrative data from a large birth cohort have higher levels of accuracy is not depending on recall (such as in retrospective surveys) and is ideal for examining risk factors over time due to the longitudinal follow-up [45]. These data—with a large N and a number of covariates—are well-suited for propensity scoring. A significant limitation (shared with almost all observational studies) is that certain covariates and mediating effects are unobservable due to lack of information. The data can only capture recorded variables; for example, only individuals seeking mental health treatment will receive a diagnosis, which may not be include all individuals with mental health conditions [46]. Sensitivity testing addresses this limitation, but such covariates might well have impacted study results. As mentioned above, not adjusting for involvement with child protective services (such as CFS) is a limitation. Although the number of teenage girls involved with CFS is relatively small, they may not be interacting with their mother or older sister on a regular basis and thus are less likely to model themselves after their family members. The availability of an educational predictor was an identified limitation. To account for the impact of educational achievement in our full cohort, educational outcomes would need to be available for everyone for grade 7 at the latest (as almost all teenage pregnancies occur after grade 7). Since educational achievement generally remains quite similar from year to year—grade 9 achievement is likely to be quite similar to grade 7 achievement [30]; this reduced odds ratio may better estimate the true odds. In several years, such variables can be incorporated into models of teenage pregnancy. Additionally, we were unable to identify Aboriginal individuals; this is a limitation as teenage pregnancy rates are more than twice as high in the Aboriginal population than in the general population [47]. Family and peer relationships, social norms, and cultural differences will likely never be measured through administrative data; limiting the degree to which these confounders can be controlled for.

## Conclusions

This paper contributes to understanding of the broader topic “who is influential about what” within the family. The teenage pregnancy risk seen in younger sisters when older sisters had a teenage pregnancy appears based on the interaction with that sister and her child; the family environment experienced by the siblings is quite similar. Much of the pregnancy risk among teenage daughters of mothers bearing a child before age 20 seems likely to result from the adverse environment often associated with early childbearing. Given that an older sister’s teenage pregnancy has a greater impact than a mother’s teenage childbearing, social modelling may be a stronger risk factor for teenage pregnancy than living in an adverse environment.

## Figures and Tables

**Fig. 4:**
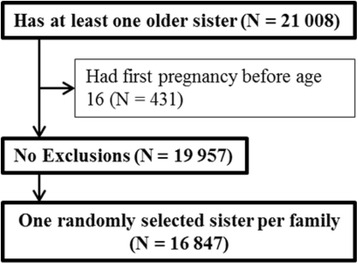
Cohort adjustment

**Fig. 5:**
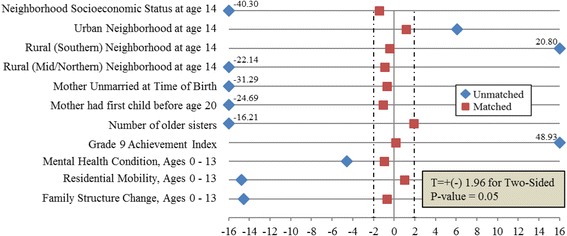
Checking covariate balance of older sister’s teenage pregnancy status

**Fig. 6:**
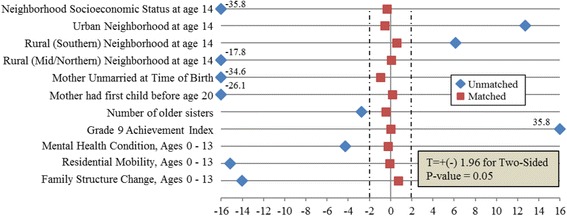
Checking covariate balance of mother’ teenage mom status

## Appendix 1

### Pregnancy diagnosis codes

Teenage pregnancy is defined as females with a hospitalization with one of the following diagnoses (MCHP, 2013):

- ○ live birth: ICD-9-CM code V27, ICD-10-CA code Z37
- ○ missed abortion: ICD-9-CM code 632, ICD-10-CA code O02.1
- ○ ectopic pregnancy: ICD-9-CM code 633, ICD-10-CA code O00
- ○ abortion: ICD-9-CM codes 634-637 ICD-10-CA codes O03-O07; or
- ○ intrauterine death: ICD-9-CM code 656.4, ICD-10-CA code O36.4

Or, a hospitalization with one of the following procedures:

- ○ surgical termination of pregnancy: ICD-9-CM codes 69.01, 69.51, 74.91; CCI codes 5.CA.89, 5.CA.90
- ○ surgical removal of extrauterine (ectopic) pregnancy: ICD-9-CM codes 66.62, 74.3; CCI code 5.CA.93
- ○ pharmacological termination of pregnancy: ICD-9-CM code 75.0; CCI code 5.CA.88; or
- ○ interventions during labour and delivery, CCI codes 5.MD.5, 5.MD.60

## Appendix 2

### Adjustment for educational achievement

To account for the impact of educational achievement on teenage childbearing, the grade 9 achievement index was adjusted for in a sub-population of individuals who had not had a pregnancy prior to age 16 (Fig. 4). As educational achievement was measured using the grade 9 achievement index (which is based on average marks in all classes and the number of credits earned during the school year [31], individuals had to have at least finished grade 9 before becoming pregnant to use this variable as a predictor.

**Table 4:** Covariates and outcomes for older sister’s teenage pregnancy status model

	Mother had first child after age 19 (*n* = 14934)		Mother had first child before age 20 (*n* = 2181)	
	Mean/proportion	SD	Mean/proportion	SD
Outcome				
Teenage pregnancy	0.094	0.292	0.372	0.483
Time invariant covariates				
Neighborhood socioeconomic status (SEFI) at age 14	−0.115	0.917	0.741	1.142
*Location of neighborhood at age 14*				
Urban	0.509	0.500	0.450	0.498
Rural South	0.278	0.448	0.132	0.338
Rural Mid/North	0.214	0.410	0.418	0.493
Mother unmarried at time of birth	0.140	0.347	0.423	0.494
Mother had first child before age 20	0.084	0.277	0.285	0.452
Number of older sisters	1.087	0.311	1.241	0.526
Grade 9 achievement score	0.214	0.934	−0.611	0.855
Time-varying covariates, ages 0–13				
Mental health condition	0.154	0.361	0.189	0.391
Residential mobility	0.555	0.497	0.689	0.463
Family structure change	0.155	0.361	0.276	0.447

### Older sister’s teenage pregnancy status

After propensity score matching, the final sample consisted of 1721 cases and 3442 controls (1:2). T-statistics were calculated for each covariate before and after matching to check for covariate balance (Fig. 5). Any point outside of the two vertical dotted lines signified a statistically significant covariate (at *p* = 0.05). All covariates differed significantly in the unmatched sample. After matching, the t-statistics of all covariates fell within the non-significant region indicating balance in cases and controls.

**Table 5:** Covariates and outcomes for mother’s teenage childbearing model

	Mother had first child after age 19 (*n* = 14763)		Mother had first child before age 20 (*n* = 2084)	
	Mean/proportion	SD	Mean/proportion	SD
Outcome				
Teenage pregnancy	0.12	0.325	0.355	0.479
Time invariant covariates				
Neighborhood socioeconomic status (SEFI) at age 14	−0.06	0.956	0.866	1.125
*Location of neighborhood at Age 14*				
Urban	0.515	0.5	0.37	0.483
Rural South	0.256	0.436	0.178	0.398
Rural Mid/North	0.229	0.42	0.432	0.495
Mother unmarried at time of birth	0.178	0.355	0.539	0.499
Older sister(s) had a teenage pregnancy	0.162	0.369	0.458	0.498
Number of older sisters	1.114	0.364	1.14	0.402
Grade 9 achievement score	0.143	0.963	−0.611	0.833
Time-varying covariates, ages 0–13				
Mental health condition	0.156	0.363	0.196	0.397
Residential mobility	0.562	0.496	0.723	0.448
Family structure change	0.16	0.367	0.309	0.462

### Mother's teenage childbearing status

After propensity score matching, the final sample consisted of 1499 cases and 2998 controls (1:2). T-statistics were calculated for each covariate before and after matching to check for covariate balance (Fig. 6). Any point outside of the two vertical dotted lines signified a statistically significant covariate (at *p* = 0.05). All covariates differed significantly in the unmatched sample. After matching, the t-statistics of all covariates fell within the non-significant region indicating balance in cases and controls.

## Abbreviations

ACG: Adjusted Clinical Group
CCI: Canadian Classification of Health Intervention
CFS: Child and Family Services
ICD-9-CM: International Classification of Diseases, Ninth Revision, Clinical Modification
ICD-10-CA: International Classification of Diseases, 10th Revision, with Canadian Enhancements
MEDC: Major Expanded Diagnostic Clusters
MCHP: Manitoba Centre for Health Policy
SEFI: Socioeconomic Factor Index
